# Impacts of Hyaluronan on Extracellular Vesicle Production and Signaling

**DOI:** 10.3390/cells14020139

**Published:** 2025-01-18

**Authors:** Melanie A. Simpson

**Affiliations:** Department of Molecular and Structural Biochemistry, North Carolina State University, Raleigh, NC 27695-7622, USA; msimpso3@ncsu.edu; Tel.: +1-(919)-515-5680

**Keywords:** hyaluronan, hyaluronidase, CD44, exosomes, microvesicles, extracellular vesicles, cancer

## Abstract

Hyaluronan (HA) is a critical component of cell and tissue matrices and an important signaling molecule. The enzymes that synthesize and process HA, as well as the HA receptors through which the signaling properties of HA are transmitted, have been identified in extracellular vesicles and implicated in context-specific processes associated with health and disease. The goal of this review is to present a comprehensive summary of the research on HA and its related receptors and enzymes in extracellular vesicle biogenesis and the cellular responses to vesicles bearing these extracellular matrix modulators. When present in extracellular vesicles, HA is assumed to be on the outside of the vesicle and is sometimes found associated with CD44 or the HAS enzyme itself. Hyaluronidases may be inside the vesicles or present on the vesicle surface via a transmembrane domain or GPI linkage. The implication of presenting these signals in extracellular vesicles is that there is a greater range of systemic distribution and more complex delivery media than previously thought for secreted HA or hyaluronidase alone. Understanding the context for these HA signals offers new diagnostic and therapeutic insight.

## 1. Introduction

Hyaluronan (HA) is an anionic glycosaminoglycan polymer that is synthesized and secreted to the extracellular space by plasma membrane-embedded hyaluronan synthase enzymes HAS1, HAS2, and HAS3 (reviewed in [1] and references therein). Newly synthesized HA ranges in molecular mass from ≈ 100 kDa to > 10 MDa and is typically found in abundance within extracellular matrices of specific adult tissues and during development [2,3]. Significant amounts of HA are continually synthesized and shed into lymphatic vessels and ultimately cleared via liver uptake in normal conditions, but elevated production and/or accumulation around cells and within tissues is reported to occur in numerous disease states (reviewed in [4,5,6,7,8,9,10,11]). HA consists of a simple linear assembly of alternating glucuronate and N-acetylglucosamine moieties and, as such, its production is subject to regulation by the metabolic status of cells and tissues [12,13]. Its retention at the cell surface and within matrices is facilitated by cell surface receptors such as CD44, matrix proteoglycans such as aggrecan and versican, and cross-linking by the TSG-6-mediated transfer of heavy chain protein from inter-α-trypsin inhibitors [14]. During active synthesis, a significant amount of HA is also retained by continued association with HAS.

The impact of excess polymeric HA is generally anti-proliferative and anti-angiogenic, and the opposite is true of HA oligomers and fragmented polymers [15]. During development and wound healing, HA is synthesized in large amounts and secreted to generate acellular matrices that promote tissue morphogenesis. HA is essential for cardiac valve development, where its rapid production initiates constriction and the movement of cellular sheets, and its regulated hyaluronidase-mediated clearance promotes both the reuptake and production of HA oligomers that serve to signal cell differentiation and create the valve [16]. Hyaluronidases are therefore also extensively implicated in cell growth, differentiation, motility, and transformation. Their aberrant function, by altering matrix composition and the context of the HA ligand that is presented to various HA receptors, is linked to numerous disease states [3,5,17].

**Figure 1:**
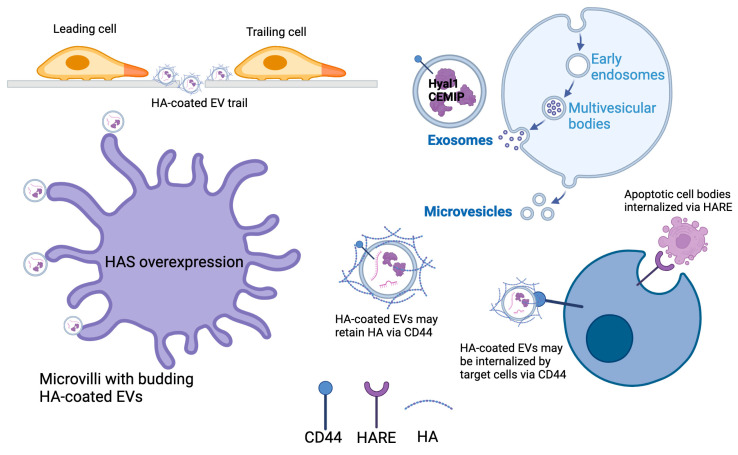
Formation of EVs and the modes of possible incorporation for HA, HA receptors, and HA metabolic enzymes. Cells that overexpress HAS synthesize and shed HA-coated EVs through the increased density of microvilli. HA and HA-coated EVs shed by one cell can generate a migration path for other cells. In some cases, HA was bound to the surface of EVs by CD44, which may also be a receptor on target cells that mediates EV docking. Hyaluronidase enzymes may be secreted and bound to HA on microvesicles, or they may be encapsulated within exosomes that are generated intracellularly in multivesicular bodies before being shed from the cell via exocytosis. Apoptotic cell bodies are also termed EVs, and their clearance from tissues may be facilitated by HA-scavenging endothelial receptors such as HARE (Created in https://BioRender.com, accessed on 23 December 2024).

Studies in the past ten years have shown that HA, along with its metabolic enzymes and receptors, can also be detected in extracellular vesicles (EVs) [18]. EVs are characterized and classified according to the size and route of biogenesis [19], but all consist of cellular materials that are encapsulated within a membrane bilayer (Figure 1). Currently, the three main classes of EVs are termed exosomes (average diameter of 50–200 nm), microvesicles (>100 nm up to ≈1 mm), and apoptotic bodies formed during cellular fragmentation (≈1–5 mm). Exosomes are formed within multivesicular bodies contained in the cytoplasm and are shed from the cell by exocytosis when the membrane of the surrounding vesicle fuses with the plasma membrane in a well-coordinated process dependent on cytoskeletal interactions, small GTPases, and endosomal sorting complexes required for transport (ESCRT family proteins). Microvesicles bud directly from the plasma membrane and are pinched from the surface by a different set of contractile proteins. In general, this review uses the term EV for all vesicle types except when specifically designated.

It is well accepted that EVs have a role in normal cellular communication in most eukaryotes and that their production is dramatically increased in diseases such as cancer and in conditions involving cellular nutrient stress [20,21,22]. The association of HA, hyaluronidases, and receptors such as CD44 with EVs has interesting functional implications for cancer and disease progression [18]. In addition, novel insights about EV biogenesis have been suggested by examination of the connection to HA metabolism. These studies are the subject of this review.

## 2. Detection of HA in EVs

EVs have a significant role in cellular interactions and cell transformations that are component processes in disease progression. For example, exosomes derived from tumors were shown to impact the metabolism of patient-derived stromal cells that caused them to attain a phenotype hallmarked by the expression of smooth muscle actin, desmin, and vimentin, as well as elevated lipid metabolism, similar to the reactive stroma found in clinical specimens [23]. Cellular transformation initiated by vesicular communication is capable of being transmitted by fluids throughout the body so the implication of that mode of communication in the progression of diseases such as cancer is not surprising. In fact, tumor-derived EVs have been shown in many studies to be capable of conditioning tissues through alterations in cell surface receptors and signal transducer profiles, such that tumor metastasis is promoted and accelerated (reviewed in [20,21,24]). The profiling of EVs through proteomics, RNA sequencing, and targeted methods has revealed an array of specific proteins such as tetraspanins (e.g., CD63) and components of cell adhesion such as mucins and integrins, as well as cargo such as RNA, microRNA, RNA binding proteins, mitochondrial DNA, proteases and other ECM-degrading enzymes, lipids, cholesterol, and miscellaneous metabolites (reviewed in [20,21,22,24]). Methods for isolating and characterizing EVs are summarized in Table 1.

HA biosynthetic enzymes HAS1, HAS2, and HAS3 are 50–70% identical on the amino acid level and have largely redundant functions but are differentially tissue and temporally expressed [1]. Therefore, their appearance in EVs and their influence on tissue-specific EV shedding is likely to vary accordingly. All three catalyze the synthesis and concurrent extracellular secretion of high-molecular-mass polymers of HA. The impact of HA on physical and functional properties of tissues, and regulated cell communication, was long thought to be primarily due to size-dependent interactions of free HA molecules with receptors and binding proteins [2].

The first association of HA with EVs was reported in 2013, when Rilla and colleagues overexpressed a GFP-HAS3 fusion protein and used quantitative fluorescence microscopy to show large increases in numbers of microvilli per cell [25]. The microvilli had both HAS3 protein and HA along the surface, and these HA-encapsulated microvilli shed HA-coated EVs at a high rate. Comparing among cell types with and without endogenous high HA production showed that HA-producing cells shed significantly more EVs.

All three HAS enzymes have now been found in EVs. HAS1 is expressed primarily in embryonic development but has been implicated in cellular plasticity through its expression in stem cells and the requirement for persistent high-molecular-mass HA for stem cell maintenance [26]. In that study, HAS1 was found in EVs that promoted wound healing and tissue plasticity [26]. HAS2 is critical for embryonic development [16], and its expression is regulated at the transcriptional and post-transcriptional levels. Its function is dependent on a series of post-translational modifications [27], which are discussed below for their importance in the localization of HAS2 in EVs. HAS3 is relatively ubiquitous in its expression but is higher in adults than in embryos [28]. Both HAS2 and HAS3 are significantly upregulated in association with multiple epithelial cancers, and these isozymes are the most frequently associated with the presence of HA in EVs [29].

## 3. Unique Roles of Hyaluronidases and HA Receptors in EVs

HA is processed and catabolized by hyaluronidases, which, in humans, are a family of enzymes consisting of Hyal1, Hyal2, Hyal3, Hyal4, PH20, CEMIP (Cell Motility Inducing Protein), and TMEM2 [30,31,32]. All members of the hyaluronidase family are encoded with a signal peptide that anchors the enzyme to the membrane of the Golgi system while post-translational maturation is completed and ensures accurate trafficking for secretion. Hyal2, Hyal4, and PH20 are membrane-tethered by a GPI linkage [30], and TMEM2 is a single transmembrane domain-containing protein [33]. The processing of HA polymers for internalization by cells within most tissues occurs outside the cell and requires secreted and/or extracellular GPI-anchored hyaluronidases. HA is endosomally internalized, and its uptake depends on a membrane-embedded receptor for cellular internalization of the receptor–HA–hyaluronidase complex. This has been reported for Hyal1 [34,35,36,37], Hyal2 [34,38], and PH20 [39] but has not been shown in conjunction with the chondroitinase activity of Hyal4 or with the apparently inactive Hyal3. More recently identified members of the hyaluronidase family, CEMIP (also known as HYBID and KIAA1199) and TMEM2 (now definitively shown to have hyaluronidase activity [40]), have been functionally characterized as cell surface hyaluronidases with activity in the extracellular space (reviewed in [31]), but their presence in EVs is an emerging possibility.

Several of the hyaluronidases identified in EVs have unique functions in this context that have been characterized in a few studies (functional results involving EVs containing HA components are summarized in Table 2). Hyal1 is largely understood as a secreted and a lysosomal enzyme [41] with activity in an acidic pH range [42], but it has been reported in EVs that were generated and shed via the exosome biogenesis route [36]. Hyal2 is associated with lipid rafts [38], which are well-documented sites of EV shedding [20]. Though Hyal2 has not been reported in EVs, its localization within lipid rafts and its known association with CD44 and Hyal1 in specifically constituted membrane lipid domains [34] suggests that it is a likely component of EVs, alongside other extracellular matrix-degrading enzymes like Hyal1, cathepsin, ADAMs, and various MMPs (reviewed in [43]). Hyal3 has also not been reported to be associated with EVs and appears to have generally cytosolic distribution despite its signal peptide, but knockout mouse and cellular functional studies suggest that Hyal3 may have a role in tissue-specific Hyal1 activity that could promote its release in some types of EVs [44].

CD44 and Hyal2 were found a number of years ago to be required for the efficient uptake of extracellular HA [34]. In this study, Hyal1 was also required, as indicated by a loss of uptake when Hyal1 expression was knocked down. In another study, Hyal1 secretion to the extracellular space was shown to be required for both the uptake of HA and the increased vesicle trafficking of chondroitin sulfate (CS)-modified surface receptors, including the recycling of receptor-containing endosomes back to the cell surface [35]. The conclusion was that Hyal1 binding to both HA and to surface CS moieties on proteoglycans was occurring in the extracellular space where Hyal1 activity was pH inhibited and that, somehow, this complex was presented to the CD44-Hyal2 complex to promote endosomal internalization. Neither study considered EVs because there had not yet been a precedent for HA and hyaluronidase presence in EVs. However, it is likely that a similar mechanism may be involved in promoting EV uptake with HA and Hyal1 present.

A significant number of studies have revealed the presence of HA receptors in EVs, finding that their differential expression was associated both with the elevated cellular production of EVs and the target cell response to receptor-containing EVs. In general, HA is assembled and retained at the cell surface of many different cell types, partly through the action of CD44, which is a single transmembrane domain-containing protein originally characterized as the primary HA receptor [45]. The retention of HA in the surrounding tissue is accomplished through extracellular aggregating proteoglycans that organize matrices such as that within cartilage in a tissue-dependent manner. HA binding receptors other than CD44, including LYVE-1 and RHAMM, have not been explicitly associated with EVs. However, a recent proteomic analysis of EVs from brains of aging mice revealed significant multi-fold increases in HA content and levels of HA and Proteoglycan Link Proteins HAPLN1 and HAPLN2 in EVs from the aged mice [46]. Neurocan, brevican, and aggrecan were also seen to increase in specific regions of the aged brains.

CD44, through binding to HA, mediates cell–cell and cell–matrix adhesion in multiple cell types [47,48]. Though monovalent with respect to its HA binding domain, the minimum binding unit for CD44 was shown to be a hexasaccharide [49]. Given the dimensions of the HA binding domain relative to the HA polymer, CD44 is capable of multivalent binding on the order of a hundred or more copies per HA strand, which transduces different signaling effects through its intracellular associations with cytoskeletal adaptor proteins and secondary messengers (reviewed in [50]). Not surprisingly, CD44 appears to mediate the HA-dependent interactions of EVs and target cells. However, how EV association impacts CD44-HA valency has not been reported.

Using correlative light and electron microscopy imaging to detect HA, HAS3, and CD44, melanoma-derived EVs of various sizes were visualized while attached to the surface of normal human keratinocytes and breast cancer cells in vitro [51]. The treatment of cells with HA 10-mers reduced numbers of adherent EVs by over 40%, suggesting at least a partial role for CD44 in adherence of EVs to the cell surface. Additional studies found a CD44-HA role in the interaction between colorectal carcinoma (CRC)-derived EVs and monocytes [52]. In the colon, monocyte CD44 promoted tumor EV uptake [53]. CD44 was further shown to retain pericellular HA on filopodia and HA-coated EVs, which was associated with the induced tumorigenicity of gastric carcinoma cells [53]. The proteomic profiling of mucosal EVs from normal and colon tumors by LC-MS/MS revealed that high CD44 in tumor cells correlated with more EVs, and CD44 intensity marked CRC cells with differential capacity for EV release [54].

CD44 coordinates activity of other HA metabolizing enzymes such as hyaluronidases. As mentioned above, the formation of a CD44-Hyal2 complex was necessary to internalize extracellular HA-Hyal1 complexes through endosomal uptake [34]. In addition, CD44 knockdown released Hyal2 to the conditioned media of primary human osteoarthritic chondrocytes [55]. CD44 and Hyal2 co-immunoprecipitated and co-localized in vesicles that were both intracellular and invaginating at the plasma membrane.

Interestingly, proteomic profiles of EVs from tumors with different properties have also revealed the presence of CEMIP. In mucosal EVs from normal tissue and colon tumors analyzed by LC-MS/MS, CEMIP was specifically found in tumor-derived EVs [56]. Exosomes from brain-metastatic tumors have been shown to condition the remodeling of the brain microenvironment to create a favorable site for metastasis [57]. Proteomic analysis showed that CEMIP was specifically elevated in exosomes from brain metastatic tumor cells and not those from cells with metastatic tropism for other tissues such as lung. Brain slices pretreated with exosomes from brain-metastatic breast cancer cells (but not those from lung or bone tumor cells) had more tumor cell growth on them when exposed in culture [57]. Proteomic analysis confirmed the presence of CEMIP in the tumor-generated EVs.

Other known members of the hyaluronidase and HA binding protein families have functional characteristics that are consistent with an active role in EV properties. In particular, TMEM2 has not been examined explicitly for presence in EVs. However, TMEM2 was found in urinary EVs of PKD1 mutant polycystic kidney disease patients [58]. This study extends the connection between TMEM2 and polycystin, which is a driver of kidney disease, highlights TMEM2 EVs as having novel diagnostic potential, and may indicate a functional role for TMEM2 in progression of the disease.

## 4. Impact of HA on EV Biogenesis and Cargo Sorting

EV biogenesis occurs via multiple mechanisms that are impacted by HA and the components of its signaling and metabolism. As discussed, microvesicles bud from sphingolipid, cholesterol-rich rafts on the plasma membrane surface that are pinched off by the sliding of actin and its associated cytoskeletal partners. These are potential sites of association with HA and HA binding proteins. Exosomes are generated intracellularly in multivesicular bodies, and the cargo, sometimes targeted by ubiquitination, is sorted at the cytosolic surface through the actions of proteins such as the endosomal sorting complexes required for transport (ESCRTs). These vesicles often contain matrix remodeling enzymes and, among them, several hyaluronidase isozymes.

Both HAS2 and HAS3 have been shown to stimulate EV shedding when overexpressed, and enzymatic activity of the HAS isozyme is critical for this function. For example, known HAS2 post-translational modifications such as ubiquitination, phosphorylation, and O-GlcNAcylation were shown to be important for the EV localization of HAS2, likely due to the role of these modifications in maintaining high levels of HAS2 cell surface expression [29]. These authors reported that the production of HA was not needed for continued shedding, but the modifications to the HAS protein may have served to maintain its continued plasma membrane location and supported the persistence of the actin-dependent microvillar structures needed to facilitate EV shedding.

Using a collagen gel overlaid on monolayer cell cultures facilitated the time lapse image capture and the subsequent quantification of microvesicles as they were shed by breast tumor cells [25]. The production of microvesicles was blocked by glucose deprivation or overnight culture with 4-methylumbelliferone (4-MU) to scavenge HA precursors and reduce HA synthesis, and the removal of 4-MU restored rapid microvesicle release. This study demonstrated that HA synthesis was directly promoting microvesicle biogenesis in a nutrient-sensitive manner, consistent with a need for ongoing HA synthesis to maintain shedding. Immunoblot analysis of the microvesicles showed they contained HAS3 and transiently produced HA. These EVs also contained actin and CD44, but HA hexamers did not dislodge the HA from the EVs. This suggests that the ongoing HA synthesis may serve to maintain the clustering of bound CD44 and/or other HA receptors to preserve their internal association with actin filaments that structurally contribute to the formation and shedding of EVs.

Subsequent studies compared endogenous HAS3 expression and HA production between isogenic malignant and nonmalignant breast tumor cell lines [59]. Malignant cells were found to express an order of magnitude more HAS3, leading to the similar overproduction of HA, which formed a pericellular matrix on the cell surface and resulted in deposits of HA that corresponded to patterns of cell motility, resembling a cellular “trail”. These cells manifested filopodial actin-containing protrusions visualized by SEM [59]. Using correlative light and electron microscopy, the authors were able to visualize and co-localize HAS3 expression, HA vesicle shedding, and the association of the vesicles with neighboring cells after microvillus-mediated contact with HAS3-overexpressing cells [60].

It has been reported that CD44 may affect multi-drug resistance (MDR, also known as P-glycoprotein, Pgp1) protein expression and function via EV-mediated transfer. CD44, which is differentially expressed in highly metastatic versus low metastatic breast cancer cells, was shown to mediate the transfer of ezrin as cargo in EVs that also transfer MDR Pgp1 to low metastatic cells and confer therapeutic resistance [61]. This did not occur within leukemia cell populations that did not have CD44 and did not transfer MDR. Ezrin localizes to the C-terminal intracellular signaling domain of CD44 and is required for Pgp1 membrane association, which is suggested to be the mechanism by which it can facilitate EV transfer, since Pgp1 needs to be at the membrane for budding.

It is clear that the overexpression of HAS and the continuous high-level production of HA in a certain size range promote the extensive formation of microvilli, from which a high rate of EV shedding occurs [25,60,62,63]. What is not clear is whether HA-encapsulated EVs are only shed through budding from the microvillar surface or if they arise via standard biogenesis mechanisms as well and what the role is for microvillar structures in the release of exosomes from multivesicular bodies. Specifically, the impact of pericellular HA on the rate of multivesicular body exocytosis to achieve the release of exosomes has not been reported, nor has whether exosomes can become encapsulated by HA as they are being shed, simply by their passage through the pericellular HA coat.

## 5. Role of HA in EV-Mediated Cellular Communication

Prior to the demonstration of HA and other ECM components as signaling molecules distributed in vesicular form, it was observed that vesicles and the shedding of “cell fragments” occurred on the migration trail of melanoma cells during invasion in three-dimensional collagen matrices, suggesting that aggressive cells within the microenvironment were capable of directly facilitating the acquisition of invasive behavior in less aggressive cells by leaving signals [64]. Subsequently, many studies showed the effects of such signals and further implicated HA-coated vesicles in dynamic cell communication. Isogenic MCF10A (non-metastatic) and MCF10CA (metastatic) cells were compared to reveal that the metastatic cells produced significantly more EVs and deposited more HA in the wake of their trailing edge during migration in vitro. The HA trail was composed of free HA embedded with HA EVs [59]. Tumor cells coordinately migrated on the HA trails. When a leading cell left an HA trail, follower cells proceeded along the trail more frequently than was observed for cells that did not leave such a trail, and these effects were eliminated by hyaluronidase treatment. These results were further supported by observing cellular pathfinding in the in vivo context using the chorioallantoic membrane assay [59]. Thus, HA-coated vesicles may stimulate motility and provide a substrate for cancer cell movement in a manner that offers greater directional incentive for coordinated movement than HA alone, which elicits relatively non-directional migratory patterns (properties summarized in Table 2).

Multiple factors have been shown to contribute to the signaling impacts of HA-coated EVs, including the rate of HA production by the leading cells, the presence of hyaluronidases and/or HA receptors within the EVs, and differential cargo contents of EVs with significant HA. In melanoma, HAS3 overexpression induced EV shedding, and EVs isolated specifically from the cells overexpressing HAS3 were shown to trigger the Indian Hedgehog (IHH)-mediated upregulation of c-Myc in target cells, leading to the transformation of those cells [51]. In prostate tumor cells, the overexpression of the hyaluronidase Hyal1 promoted accelerated vesicle trafficking through endosomal internalization and recycling, leading to increased rates of cell proliferation and motility [35]. Tumor cells containing Hyal1 produced more EVs and grew more quickly into three-dimensional spheroids from which cells emerged more rapidly on collagen [36]. The treatment of prostate stromal cells with tumor-derived EVs containing Hyal1 specifically promoted stromal cell motility via FAK and β1 integrin on the stromal cells [36]. Thus, EVs containing the HA processing enzymes offer a way for cells to deposit and localize the signaling potential of HA for acquisition by other cells.

The effect of HA receptors on signaling potential and mechanisms has also been examined. In ovarian cancer cells, EVs were shown to be coated with CD44, which mediated mesothelial cell uptake [65]. Moreover, high CD44 in peritoneal mesothelial cells increased MMP9 and thereby facilitated invasion of the ovarian tumor cells. Another study in ovarian cancer provided evidence that exosomes from highly metastatic cells in a heterogeneous tumor population could transform those cells with low metastatic potential and increase the rate of tumor progression. The authors found that CD44 transfer via EVs from cells with high CD44 to cells with low CD44 increased migration and invasion of the latter [66]. In CRC cells, high CD44 levels led to the enhanced shedding of EVs, which was associated with organoid formation and increased cell proliferation. However, although CD44 intensity appeared to be a marker of cells with differential capacity for organoid formation and EV release, the authors showed that it does not always appear to guide cargo selection [54]. Further studies have suggested chemotherapy (e.g., paclitaxel) could induce CD44 upregulation. This treatment in breast cancer cells resulted in increased CD44-positive EV shedding that was associated with reduced adhesion and cell spreading, favoring tumor cell invasion [67]. Although not explicitly measured in this context, those metastatic breast cancer cells also have high levels of HAS expression and HA production [68].

Several recent studies have examined the role of EVs in promoting invasive cancer and metastasis in the brain. Glioblastoma cells with mutant p53 develop a proinvasive phenotype [69]. The authors found that these cells had significantly increased expression of the sialomucin podocalyxin, which could be released to the brain via EVs. When astrocytes in brain slices were exposed to podocalyxin-containing EVs from the glioblastoma cells, they were induced to make HA, which in turn caused the glioblastoma cells to migrate robustly onto these treated slices. Interestingly, a study of brain metastatic tumor mechanisms found CEMIP highly expressed in brain metastases, where CEMIP-containing EVs were shown to precondition the metastatic niche [57]. CEMIP-expressing metastases showed evidence of vascular co-option, presumed to enable tumor cells to cross the blood–brain barrier, and the invasion of brain slices was enhanced by CEMIP EVs but not EVs from cells in which CEMIP expression was knocked out. Mice injected intracardially with CEMIP wild-type cells had significantly more brain metastases than those injected with CEMIP knockout cells. Importantly, this effect could be reproduced with CEMIP knockout tumor cells if mice were preinjected with only CEMIP wild-type EVs. Both endothelial and glial cells internalized EVs with CEMIP, and this induced the branching of endothelial cells in the perivascular space via proinflammatory cytokines, thereby promoting colonization by brain-metastasizing tumor cells [57].

Another recent study examined the impact of tumor-derived HA-containing EVs on immune function in the context of pancreatic ductal adenocarcinoma (PDAC) progression [70]. It was found that the PDAC cells produced EVs carrying HA, had higher levels of HA synthesis pathway enzymes, and the expression of these enzymes correlated with worse survival rates. The hexosamine biosynthesis pathway is more active in PDAC than normal cells, fueling HA synthesis. It is known that HA-rich stroma in PDAC contributes to therapeutic (gemcitabine) inefficacy and resistance and that combined treatment with gemcitabine and hyaluronidase extends progression-free survival [71]. However, the study further reported that HA-containing EVs activated and differentiated monocytes, which produced IL-1β and increased stellate cell MMP-9 to promote invasion [70]. Activated stellate cells promoted immunosuppression by the release of multiple cytokines that led to T cell exhaustion. By causing stromal HA accumulation, these HA EVs were protumorigenic through immune suppression (reduced CD8+ T cells). Analysis of data in The Cancer Genome Atlas revealed that GFAT1/2 (glutamine-fructose-6-phosphate amido-transferase, the rate-limiting enzyme in the hexosamine biosynthesis pathway, which generates one of the two HA precursors) along with HAS2 and HAS3 were significantly elevated in PDAC relative to healthy patients. The inhibition of GFAT or scavenging HA precursors both reduced HA in EVs by 30–60% in PANC1 and AsPC1 pancreatic tumor cell lines.

Finally, circulating HA, other glycosaminoglycans, and HA-encapsulated EV ligands such as apoptotic cell bodies have been shown to clear through the liver and lymphatics via the hyaluronan receptor for endocytosis (HARE, also known as Stabilin-2 [72]). HARE is expressed on sinusoidal endothelia of liver, lymph nodes, and bone marrow [73,74], and the binding of its diverse GAG ligands triggers ligand–receptor uptake by endocytosis, leading to the clearance of the ligands in the case of liver and lymphatics, or signaling events in the bone marrow (reviewed in [72,75]). In addition to apoptotic cell bodies, HARE is likely to have a role in the clearance of other types of EVs (exosomes and microvesicles) from circulation, but this has not been explicitly examined. Consistent with this, an anti-HARE blocking antibody prevented lymph node metastasis following an orthotopic injection of mice with HA-encapsulated metastatic prostate tumor cells, and pretreatment of the animals prior to cell implantation was necessary to prevent metastatic invasion, suggesting HA-coated EVs responsible for HARE-dependent LN invasion were prevented from conditioning the LN niche [76].

## 6. Implications of HA for EV Elasticity and Mechanobiology

The biophysical properties of HA have been used to detect, quantify, and evaluate structural impacts on EVs in several recent studies. Using a combination of circular dichroism spectroscopy, atomic force microscopy, and atomic force spectroscopy, significantly greater numbers of EVs were identified in conditioned media from CRC relative to normal colon cells, and these EVs were found to be HA-encapsulated [77]. Further examining the HA-containing EVs by single-molecule force microscopy allowed the group subsequently to measure HA chain lengths on the EVs [78]. They reported an average HA polymer size of < 200 kDa and found that these EVs were more elastic and flexible than those lacking HA as produced by normal cells.

The significance of HA for mechanobiology and plasticity was systematically examined in a study of membrane shape regulation by the cell glycocalyx [63]. Both HA and mucin impact the presence of tubular and spherical extensions of the plasma membrane, which are seen not only in disease but in specific stages of development and in cellular functions. Curved protruding membranes increase the cellular surface area, which is important for absorption, transport, environmental sensing, secretion, pinpoint delivery of cargo between cells, antibody–antigen recognition, etc. Mucins, HA, and HAS are highly represented on cell protrusions such as the microvilli of oocytes and mesothelium [79,80], neuronal axons [81], as well as tumor cells, enterocytes, astrocytes, and dendritic cells. All cell types with abundant mucin, HA, and other glycocalyx components also have extensive microvilli and secrete a lot of EVs. Shurer et al. [63] described the substructure as resembling a “brush”, which can be explained by a molecular crowding theory predicting that steric interactions and reduced available molecular configurations cause pressure on the cellular surface and result in deformations of the lipid membrane (e.g., [82]).

In an elegant and rigorous examination, Shurer et al. [63] used a genetically encoded library of native semi-synthetic and rationally designed heavily glycosylated mucin polymers that varied in size, sequence, and membrane-associated region, which were fused to the transmembrane domain of mucin. All constructs were seen to cause dramatic protrusions of tubules from the membrane that were eliminated by enzymatic digestion of the mucin. These observations were further reproduced by overexpressing HAS3 to induce the overproduction of HA. The authors validated results of ectopic HAS3 expression using primary isolated equine synoviocytes, which have high HAS3 and HA and a highly tubulated cell surface that was eliminated by hyaluronidase. Thus, the HA glycocalyx has a significant role in stabilizing the protrusions that support membrane vesicle production, also reduced by hyaluronidase, or by the depolymerization of actin filaments in the tubules. Moreover, the disruption of O-glycan extensions on components of the glycocalyx by knocking out precursor transport via SLC35A1 also reduced density of the tubules. The authors concluded that the dense glycocalyx caused an entropic bending force that favored curved membranes and supported energetics of tubule and vesicle biogenesis.

## 7. Diagnostic and Therapeutic Implications of HA-Containing EVs

Numerous reviews have comprehensively addressed the diagnostic and therapeutic potential of EVs for cancer and other diseases, and this is a rapidly emerging area of research [18,20,21,22,24,43,83,84,85]. Since HA, hyaluronidases, and HA receptors have been shown to be differentially present (usually elevated) in EVs from diseased cells and tissues, these components are obvious disease biomarkers. Here, several recent advances in potential uses of these EVs in diagnosis and therapy are summarized.

Both chemotherapy and radiation therapy have been associated with the promotion of EV shedding by tumor cells, in which HAS expression was induced, resulting in HA encapsulation of the EVs. The implication of this process is that antagonizing HA production or signaling concurrently with chemotherapy and radiation may improve the efficacy of these treatments [67,86]. HA production has been shown to be reduced in multiple contexts by 4-methylumbelliferone (4-MU), which scavenges the UDP-glucuronate precursor [70,87,88,89]. In pancreatic cancer, both the 4-MU treatment and inhibition of hexosamine precursor biosynthesis by diazo-oxo-norleucine (DON) reduced HA-coated EV production by tumor cells and resulted in the reduced secretion of protumorigenic cytokines by pancreatic stellate cells exposed to the EVs [70]. DON, as a glutamine analog, is not a specific inhibitor of HA synthesis, but the rate-limiting enzyme of the hexosamine biosynthesis pathway is among the anti-tumorigenic targets reported for this compound. Thus, the suppression of HA production may enhance the efficacy of tumor immunotherapy approaches as well.

Understanding the role of HA and CEMIP in brain cancers and brain metastasis has emerging significance for therapeutic strategies. In a study conducted by Rodrigues, et al. [57], CEMIP expression in primary tumors of brain metastasis patients predicted brain metastatic progression, and EVs in the blood of patients with brain metastasis had more CEMIP. CEMIP is involved in memory and synapse formation [90] and specifically engages the unique form of angiogenesis in the brain, termed intussusceptive angiogenesis, in which the host tissue angiogenic process is incorporated into growth of the metastatic lesion. Vessel morphogenesis was a top process altered in the brain endothelial cells by CEMIP [57]. In another study, the technique of glycan node analysis, which is a mass spectrometry-based glycomic method, was used to detect hexoses and hexosamines and compare the presence of HA on EVs from brain metastatic and non-metastatic melanoma cell lines [91]. Metastatic cells generated more EVs, which was previously correlated to worse survival. Interestingly, however, these authors found 3-fold more glucosamine in the non-metastatic cell-derived EVs, attributable to the abundance of HA. Brain-metastatic EVs contain CEMIP, which promotes the internalization and degradation of HA and may account for lower surface HA in these EVs. Also, the presence of HA in brain tumors may result from the glial cell induction of astrocyte HAS activity [69], so the HA within the brain may actually recruit CEMIP-containing EVs to condition the niche for tumor invasion. This axis has both prognostic and therapeutic potential.

## 8. Summary and Conclusions

From the initial observation that the overexpression of HAS3 could result in the production of EVs coated with HA to multiple mechanistic studies on the role of HA and its synthesis, processing, and signaling in the EV context, key functional roles of this axis in tissue-specific consequences of EV exposure have emerged: (1) High levels of HA production drive cell surface changes that lead to vesicle budding and increased rates of EV shedding. (2) EVs coated with HA have receptor-mediated impacts on target cell signaling. (3) EVs containing HA receptors and/or HA matrix remodeling enzymes can trigger significant alterations in the architecture of the pre-metastatic niche.

As a result of the many studies of the EV context for HA, it is possible that some previously published research on HA functions may bear revisiting to consider whether alternative interpretations arise. For example, in the examination of studies on exosome-mediated metastasis, there are some studies that show EVs promote metastasis and some that find they suppress or inhibit metastasis (reviewed in [92]). This raises the question of whether there is a threshold for EV numbers required for metastatic promotion and if this is cargo-dependent, with greater metastatic efficiency if more tumor-promoting cargo is presented. It has been previously seen that there is a threshold for HA production and its effects on tumorigenesis, where HA in the tumor microenvironment has been reported to accelerate tumorigenesis unless its rate of production leads to the growth-suppressive accumulation of HA [93,94,95]. It is interesting to contemplate that there may be a biophysical correlation with the presence of HA on EVs or the induction of HA synthesis in the target tissue, altering conditions for tumor cell invasion.

It is worth noting that care must be taken to avoid contaminants in EV preparation and that HA has been identified as a contaminant of certain EV isolation procedures [96]. In particular, HA in the range of ≈ 200 kDa was found in EVs isolated by methods that are strictly dependent on fluid-based separation (e.g., size exclusion chromatography, etc). These methods are used because they are less compromising to the integrity of the EVs and allow for higher throughput in handling, but EVs used in functional studies need to be evaluated for the potential complicating presence of free HA.

Additional questions that may be of interest include whether CEMIP and other hyaluronidases are primarily packaged as cargo in EVs shed through the exosomal route or if they can be noncovalently associated at the EV surface by binding to HA. An intravesicle location implies that access to target cells is dependent on other docking ligands on the EV surface. In addition, it will be interesting to look for specific effects of HA on EV contents. HA receptors and/or hyaluronidases may impact cell-specific cargo, or the route of biogenesis to favor either exosome or microvesicle shedding. CD44 has been shown in some studies to support the adhesion of HA to the EV surface, but it may also serve as a counter-receptor on the target cells to dock the HA-decorated EVs on the cell surface. It is not known whether the vesicle curvature (e.g., exosome versus microvesicle) or size would affect the apparent valency or strength of CD44-HA binding. Understanding these properties is important for therapeutics that seek to use synthetic EV-mimetic nanoparticles.

## Figures and Tables

**Table 1:** Summary of methods used to isolate and characterize EVs.

Method	Principle	Use	Limitations
**Methods of isolation**			
**Differential centrifugation**	Cells and large organelles pellet at higher speeds	Separate cell debris from organelles and vesicles	Macromolecular aggregate contamination
**Density gradient centrifugation**	Organelles separate by buoyancy	Isolate exosomes, microvesicles, organelles with high purity	Time consuming, vesicle integrity may alter during isolation
**Affinity capture**	Magnetic beads coupled to an EV biomarker	Select EVs specifically from complex mixture based on surface biomarkers	Appropriate biomarker is difficult to identify
**Size exclusion chromatography**	Organelles and EVs separated by size in flow process	Fluid-based separation without mechanical handling	Similarly sized macromolecular contaminants
**Methods of characterization**			
**Microscopy**	Fluorescence	Visualize or localize labeled cell components	Resolution insufficient for subcellular detail
	Electron (SEM, TEM)	Highest resolution	Damaging sample preparation
	Correlative light and electron	Colocalize labeled component with subcellular structure	Combines resolution, molecular detail
**Atomic force measurements**	Atomic force microscopy	Visualize 3D cell landscape at nm resolution	No molecular identification
	Atomic force spectroscopy	Measure strength of binding interactions	Surface material measurement dependent on cantilever probe
**Nanoparticle tracking analysis**	Differential light scattering of particles in liquid	Average particle diameter	Does not distinguish contaminants
**Immunoblot**	Protein detection	Specific protein identification	Specificity
**Proteomics**	Global protein analysis	Protein cargo	Does not distinguish contaminants, subset of proteins
**RNA sequencing**	Global RNA analysis	RNA cargo	Only measures RNA
**Glycan node analysis**	Global glycan determination	Quantify monosaccharides in the EV glycome	Only measures glycans
**Functional assays**	Proliferation	EV impact in vitro	Outcome depends on EV quality
	Motility/invasion	EV impact in vitro	Outcome depends on EV quality
	Tumorigenesis/metastasis	EV impact in animal models	Outcome depends on EV quality

**Table 2:** Summary of EV results featuring HA, hyaluronidases, and HA receptors.

Component	Analysis Context and Methods	Outcomes	Cells/Tissues
**Hyaluronan (HA) and HAS**			
**HA**	EVs of 150–200 nm, likely microvesicles Isolated by differential centrifugation Validated by hyaluronidase sensitivity, NTA, EM, fluorescence confocal	HA-coated EV shedding Microvilli surface density Directed motility Growth, invasion, metastasis Immune suppressive HA EVs elevated in brain of aging mice	Ovarian, breast tumor cell lines Brain slices Pancreatic cancer mouse model Primary astrocytes and glial cells Chorioallantoic membrane
**HAS1**	Centrifugation Validations: NTA, EM, Western blot	Cell plasticity, stem cell maintenance, wound healing	Human umbilical cord mesenchymal stem cells
**HAS2**	Centrifugation Validations: NTA, EM, Western blot, 4-MU sensitive	EV shedding, microvilli	COS1 transfectants
**HAS3**	Centrifugation Validations: NTA, EM, fluorescence, Western blot, 4-MU sensitivity	EV shedding, microvilli Directed motility Cell transformation	MDCK, breast tumor cell lines (multiple)
**Hyaluronidases**			
**Hyal1**	Centrifugation Validations: NTA, EM, Western, fluorescence confocal	EV rate of release and vesicle trafficking increased Cell proliferation and motility Cell transformation metastasis	Prostate tumor and stromal cell lines Mouse model Esophageal cancer
**Hyal2**	Western blot, lipid rafts	HA internalization Inflammatory bowel	Colocalizes with CD44, cells and mice
**PH20**	Centrifugation Western, NTA	Dendritic cell maturation, anti-tumor immunity	HEK293T transfectants Mouse model
**CEMIP**	Centrifugation Western, NTA	Brain metastasis Niche preconditioning Proliferation and motility Vascular co-option in brain	Brain metastatic tumor cells (breast, colon/CRC) Mouse models and primary brain slices
**TMEM2**	Differential and density gradient centrifugation Western, proteomics	Polycystic kidney disease EVs, biomarker	Human urinary EVs
**HA receptors**			
**CD44**	Centrifugation Western, NTA, EM	EV shedding, transfer of CD44, HA retention on EVs Target cell docking of EVs Invasion, transformation Metastasis, drug resistance	Breast, ovarian, CRC, gastric cancer cells Mouse models
**HARE**	Apoptotic cell bodies prepared by differential centrifugation, validated by Western	Clearance receptor for EVs in liver, lymph node, bone marrow endothelium Lymph node metastasis	Prostate tumor cells in mouse model

## Data Availability

Not applicable.
